# Pediatric Visceral Leishmaniasis in Albania: A Retrospective Analysis of 1,210 Consecutive Hospitalized Patients (1995–2009)

**DOI:** 10.1371/journal.pntd.0000814

**Published:** 2010-09-07

**Authors:** Raida Petrela, Loreta Kuneshka, Eli Foto, Ferit Zavalani, Luigi Gradoni

**Affiliations:** 1 Pediatric Department, University Hospital Center “Mother Theresa”, Tirana, Albania; 2 Institute of Public Health, Tirana, Albania; 3 Unit of Vector-Borne Diseases and International Health, Istituto Superiore di Sanità, Rome, Italy; Institute of Tropical Medicine, Belgium

## Abstract

**Background:**

Little information is available about infantile visceral leishmaniasis (VL) in Albania as regards incidence, diagnosis and management of the disease.

**Methodology/Principal Findings:**

Demographic data, clinical and laboratory features and therapeutic findings were considered in children admitted to University Hospital of Tirana from 1995 to 2009, and diagnosed as having VL. The diagnosis was based on bone-marrow microscopy/culture in 77.5% of patients, serology in 16.1%, and *ex juvantibus* in 6.4%. A total of 1,210 children were considered, of whom 74% came from urbanized areas. All patients were in the age range 0–14 years, with a median of 4 years. Hepatosplenomegaly was recorded in 100%, fever in 95.4% and moderate to severe anemia in 88% of cases. Concomitant conditions were frequent: 84% had bronchopneumonia; diarrhea was present in 27%, with acute manifestations in 5%; 3% had salmonellosis. First-line therapy was meglumine antimoniate for all patients, given at the standard Sb^v^ dosage of 20 mg/kg/day for 21 to 28 days. Two children died under treatment, one of sepsis, the other of acute renal impairment. There were no cases of primary unresponsiveness to treatment, and only 8 (0.67%) relapsed within 6–12 months after therapy. These patients have been re-treated with liposomal amphotericin B, with successful cure.

**Conclusions:**

Visceral leishmaniasis in pediatric age is relatively frequent in Albania; therefore an improvement is warranted of a disease-specific surveillance system in this country, especially as regards diagnosis. Despite recent reports on decreased responses to antimonial drugs of patients with Mediterranean VL, meglumine antimoniate treatment appears to be still highly effective in Albania.

## Author Summary

Albania is a developing country that is rapidly improving in social, economic and sanitary conditions. The health care system in still in progress and the impact of some infectious diseases remains poorly understood. In particular, little information is available on incidence, clinical features and response to treatment of visceral leishmaniasis (VL) in childhood. We performed a retrospective analysis of data recorded from 1995 to 2009 at the national pediatric reference hospital of Tirana where any child suspected for VL is referred for specific diagnosis and treatment. Epidemiology, clinical features and management of the disease were considered. The main findings can be summarized as follows: i) The incidence of the disease in Albanian children (25/100,000 in the age group 0–6 years) is much higher than in developed Mediterranean countries endemic for VL; ii) The disease is associated with poor sanitary conditions as suggested by the high rate of severe clinical features and frequency of co-morbidities; iii) The cheapest drug available for Mediterranean VL treatment (meglumine antimoniate) is highly effective (99% full cure rate) and well tolerated. Limitations were identified in the low standard laboratory diagnostic capability and unsatisfactory medical surveillance in less urbanized areas. An improvement is warranted of a disease-specific surveillance system in Albania.

## Introduction

Zoonotic visceral leishmaniasis (VL) is a disseminated protozoan infection transmitted by phlebotomine sandflies, caused by *Leishmania infantum* in areas of the Old and New Worlds [1]. In Mediterranean countries, about 1,000 people are estimated to be affected by clinical disease annually [2] although asymptomatic or sub-clinical cases are by far more frequent [3]–[5]. Mediterranean VL affects primarily children as well as an increasing rate of immunocompromised and immunosuppressed adult individuals, such as HIV- infected [6] and patients under any immunosuppressive therapies [7]–[8]. The disease is known to occur in Albania since 1938 typically as a childhood disease [9]; however, despite being a notifiable disease in the country, VL case records and statistics have not been available to international health organizations (such as World Health Organization) nor to the scientific community for long time. Albania is a developing country that is progressively increasing its social, economic and sanitary relationships with western countries. However, being its health care system still in progress, there are incomplete data on the clinical epidemiology of some infectious diseases. In particular, little information is available about VL in children as regards disease diagnosis and management. Herein we present the data derived from an observational retrospective cohort study performed at the University Hospital “Mother Theresa” of Tirana (UHT), aimed at the analysis of the epidemiological, clinical, diagnostic and therapeutic features of pediatric VL in Albania in the 1995–2009 period.

## Methods

### Study design, diagnosis and treatment

We evaluated the data regarding 1,210 children admitted from 1995 to 2009 to the Infectious Diseases ward of UHT, the largest pediatric hospital of Albania (about 400 beds). The ward (45 beds) is the national reference centre where any Albanian children suspected or diagnosed for VL in peripheral hospitals are referred for diagnosis and/or treatment. Demographic, clinical and laboratory findings were collected prospectively into a database and the data analyzed retrospectively by the UHT medical staff (RP and LK).

Diagnosis and therapy approaches followed systematically the guidelines for VL management approved by the Ministry of Health and adopted by the sanitary directorship of UHT. They include the following general examinations: full hematologic assessment, biochemical profile (serum urea, creatinine, ALT, total proteins, protein electrophoresis and bilirubin), electrolytes (K+), hemoculture, urine examination, ECG, chest x-ray and abdominal sonography. Among infectious diseases, differential diagnosis includes brucellosis, abdominal typhus and HIV.

First-line VL diagnosis was based on the microscopic demonstration of *Leishmania* amastigotes on Giemsa-stained smears of bone marrow aspirates. Aspirate material was also seeded in culture media for *Leishmania* (NNN, Evans' Modified Tobie's or MEM media) when available from the Tirana Institute of Public Health. If direct parasitological diagnosis proved negative but strong clinical suspicion of VL persisted, search for anti-*Leishmania* antibodies was performed using ELISA or IFAT commercial kits (IgG and IgM, BioMerieux, France). This second-line serological approach has been in use starting from 1999. When serology could not be performed because of shortage of kits due to financial constraints, patient's parents or guardians were recommended to send sera to private laboratories by their own. In case border-line serology results, a new serum sample was examined after one week by two different laboratories. In patients with negative bone marrow and negative or border-line serology, but showing persisting clinical and laboratory features suggestive of VL without evidence of other systemic diseases, presumptive treatment was administered and diagnosis performed *ex juvantibus*. Upon diagnosis, the first-line treatment was administered in hospital using meglumine antimoniate once daily, given intramuscularly, at the Sb^v^ dosage of 20 mg/kg/day for 21 to 28 days. Patients relapsing following initial cure were re-treated using intravenous liposomal amphotericin B at the dose of 3 mg/kg/day in days 1–5, 14 and 21 [10]. Treatment-associated adverse events of grade 2 or higher were considered.

### Ethical considerations

Clinical samples were collected from children suspected as having VL, following the national guidelines for laboratory diagnosis of the disease at UHT. Informed written consent was obtained from parents or guardians and recorded on the medical chart before performing bone-marrow aspiration. The study was approved by the National Committee of Bioethics, Ministry of Health, Republic of Albania.

## Results

### Demography and epidemiology

**Figure 1:**
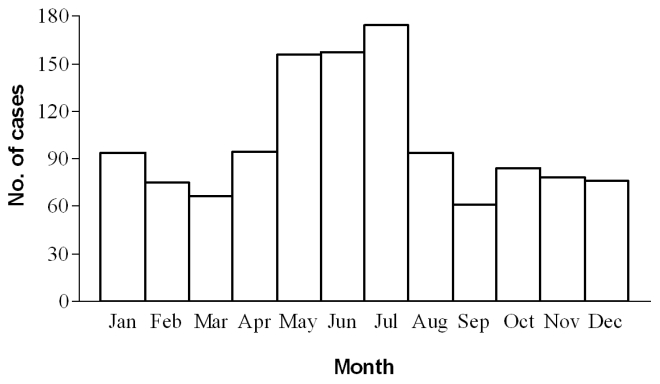
Seasonal distribution by month of diagnosis of 1210 pediatric cases of visceral leishmaniasis diagnosed from 1995 through 2009.

The yearly number of admitted patients ranged from 63 to 146, with a mean of admitted per-year of 93 patients. The monthly distribution of cases referred to the date of hospitalization and VL diagnosis is shown in the graph of Figure 1. Cases were identified in all months, with a minimum in September (61 cumulative cases) and a maximum in July (174 cases). The main cluster was in the May–July period (487 cases, 40%). Because over half of the patients referred onset of symptoms 1–3 months before hospital admittance (see below), the cluster is consistent with the usual long incubation period (around 6 months) of *L. infantum* infections transmitted late in the preceding sand fly season (May–October) [9].

**Figure 2:**
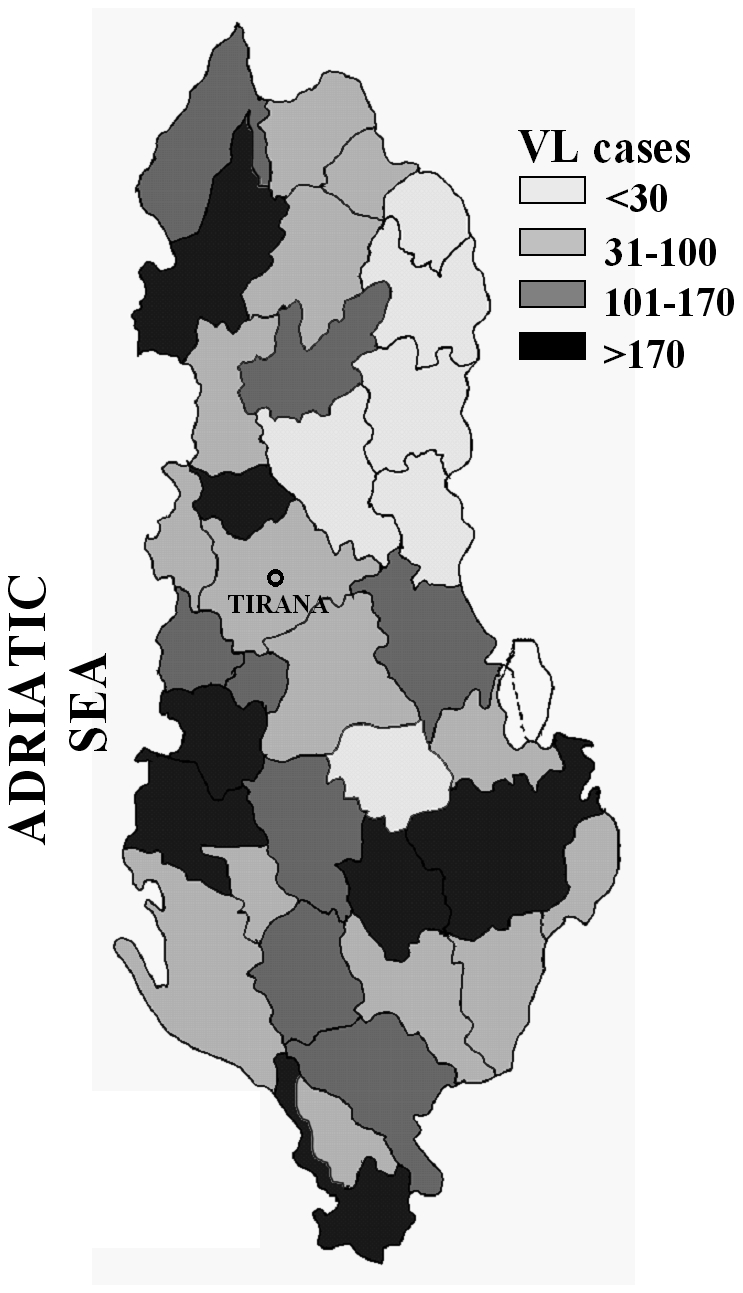
Schematic map of Albania showing the stratification by district of cumulative VL cases diagnosed in children from 1996 through 2009.

Seventy-four % of patients came from urbanized areas, mainly from peripheral districts of Shkodër and Lezhë in the north, Tirana in the centre and Lushnjë, Fier, Berat and Vlorë in the south, whereas 26% were from rural areas represented by coastal or lake territories. All districts of Albania were involved, with large variations in the distribution of cases. A map showing the stratification of cumulative cases by district is shown in Figure 2.

The age of patients ranged from 0 to 14 years (median: 4 years). The majority of cases belonged to the 1–4 years age group (61%). Sixteen % were in 0–1, 17% in 4–10 and 6% in 10–14 years age groups, respectively. Fifty-eight% were males.

### Clinical features and laboratory data

At the admittance, 42.2% of patients reported the onset of symptoms in the last 30 days or less, for a minimum of 10 days, and 57.8% since more than 30 days, for a maximum of 3 months. At presentation only 4.5% of the patients was afebrile; fever >38°C was recorded in 63.3% of patients. In Table 1 the main VL symptoms and signs of the patients and data from physical examination are summarized. Regarding hematological evaluation, the most frequent finding was anemia (99.8%) with a mean value of hemoglobin 7.0 g/dl. Hematological and biochemical features recorded at admittance are shown in Table 2. In addition, concomitant conditions were frequently recorded: 84% of patients had bronchopneumonia; diarrhea was present in 27%, with acute manifestations in 5%; 3% of patients had salmonellosis.

**Table 1:** Symptoms and signs of patients and data from physical examination.

Symptoms and signs^1^	Frequency (%)	Physical examination^1^	Frequency (%)
Fever	95.4	Splenomegaly	100
Malaise	84.9	Hepatomegaly	100
Anorexia	81.9	Abdomen enlargement	73.0
Pallor	81.9	Dry skin	28.1
Weight loss	66.8	Lymphadenopathy	11.0
Perspiration	49.2	Hemorrhagic symptoms^2^	6.5
Dry cough	29.6	Edema	2.0
Diarrhea	26.6		
Vomiting	19.1		

1 More than one symptom and sign or clinical findings at physical examination could be present in the same patient.

2 Ecchymosis, petechiae, epistaxis, or bleeding in the sites of injection.

**Table 2:** Hematological and biochemical features recorded in patients at admittance.

Finding	Frequency (%)
Anemia degree (g/dL hemoglobin)	
I (12.8 to 9.6)	11.5
II (9.5 to 6.4)	48.4
III (6.3 to 3.2)	38.4
IV (<3.2)	1.5
Reticulocytes increase above the normal range (%)	
0–15	45.1
16–30	35.4
>30	9.6
Neutropenia (<30% neutrophils)	49.0
Lymphocytosis	33.5
Lymphopenia	34.6
Thrombocytopenia (<1.5×10^5^ platelets/mm^3^)	
1.4-1×10^5^	29.6
1-0.5×10^5^	16.3
5-1×10^4^	1.4
<1×10^4^	0.7
Erythrocyte sedimentation rate (>20 mm/Hg)	95.7%
Hypergammaglobulinemia	100
Elevated globulins/albumin ratio (>1.21)	100
Hypoproteinemia (<6.5 g/dl)	13.4
Serum creatinine increase	4.8
Liver enzyme (ALT) increase	0

### VL diagnosis

In 938 patients (77.5%) the bone-marrow aspirate resulted positive for the microscopic research of *Leishmania* amastigotes. Cultures of bone marrow aspirates, performed in one fourth of the patients, resulted positive in 65% of cases, all being positive also to microscopy. Serology was performed in a limited number of bone-marrow positive patients (40), providing however some information on the validity of the commercial kits employed: 20 sera were examined by IFAT and 20 by ELISA (a retrospective evaluation included also 27 patients without leishmaniasis but affected by other systemic pathologies). Among the 272 patients in whom bone-marrow smears did not reveal amastigotes, 203 (admitted from 1999) have been examined serologically: 145 by IFAT and 58 by ELISA. Overall serology detected 195 clear positives in this subgroup, and 39/40 in the bone-marrow positive subgroup. Because there were 3 false weak positives among non-leishmaniasis patients, the mean sensitivity and specificity values of the combined IFAT and ELISA tests were estimated to be 96% and 92%, respectively. Finally, there were 69 patients with negative bone marrow that could not be examined serologically because admitted before 1999. These patients, and the 8 bone-marrow negative patients with negative/border-line serology (for a total of 77/1,210, 6.4%) have been diagnosed as affected by VL because they showed persisting clinical and laboratory features strongly suggestive of the disease, other diseases were excluded, and presumptive treatment administered resulted in clinical cure.

### Treatment

All the patients were treated with meglumine antimoniate. Two children died in course of treatment (0.16%), one for sepsis the other for acute renal impairment. There were no cases of primary clinical unresponsiveness to the first-line therapy. By the end of treatment a second bone-marrow aspirate was performed and found negative in all patients. Eight patients (0.67%) showed a clinical relapse of VL, confirmed parasitologically, within 6–12 months from therapy and needed re-treatment with liposomal amphotericin B, which successfully cured these patients as assessed by 1-year post-therapy follow-up. In 39% of the children treated with meglumine antimoniate at least one adverse event was recorded (Table 3). Patients treated with liposomal amphotericin B did not show adverse events.

**Table 3:** Incidence of adverse events recorded in 1,210 pediatric patients treated with meglumine antimoniate.

Parameter	Frequency (%)
Decrease of hemoglobin	20
Hyperamylasemia	10
Hypokalemia	7
ECG modifications	6
Hyponatremia	2
Nefrotoxicity	2
Liver enzyme (ALT) increase	1

## Discussion

Globally, there are an estimated 500,000 new cases of VL and more than 50,000 deaths from the disease each year, however both figures are approximations as VL is frequently not recognized or not reported [2], [11]. Migration, lack of control measures and HIV co-infection are the three main factors reported for driving the increased incidence of VL [12]. Poverty and leishmaniasis are also strictly associated. Poor housing conditions and diet, poverty-related concurrent infections as well as proximity to infected dogs, are all specific factors related to zoonotic VL [13]. As regards the VL transmission potential, at least 3 proven *L.infantum* vectors are present Albania, *Phlebotomus neglectus* being the most widespread and showing the highest relative density among *Phlebotomus* species [9]. Although no systematic canine surveys have been performed throughout the country, a few studies suggest that canine infections are widespread, with seroprevalence (IFAT) rates of 16–17% recorded in several districts including Tirana (Teita Myrseli, Institute of Public Health, Tirana, personal communication).

Our experience shows that VL in the pediatric age occurs frequently in Albania, with a high number of per-year admissions relatively constant during the 15-year period of observation. VL is also recorded in adults [9]: from 1998 to 2008, 126 cases diagnosed in the age groups 15–>60 years have been notified to the Control of Infectious Diseases Department, Institute of Public Health, Tirana. Furthermore, HIV-*Leishmania* co-infections have been diagnosed in 26 adult patients out of some 300 HIV/AIDS cases recorded from 1993 to 2010. These figures indicate that VL is still largely an infantile disease in Albania, the children representing 87.7% of total VL cases.

Being the surveillance system in Albania still in development we can infer that the number of actual cases including the undiagnosed or misdiagnosed ones could be even higher. In fact, a high proportion of patients came from urbanized areas, therefore small villages or farming areas might suffer from the phenomenon of under-diagnosis in rural territories with a lower degree of medical surveillance. According to the 2001 census, in Albania live about 330.000 children under 6 years (www.instat.gov.al). Based on our VL figures (90% of cases belong to this age group) we can estimate an yearly incidence of 25/100.000 of this at-risk population, which is much higher than commonly observed in southern European countries endemic for VL [2], [14].

Clinical presentation and laboratory findings reflect classic features of a poverty-related disease. The relatively high number of co-morbidities among the patients is noteworthy, as it is the elevated prevalence of severe anemia; they may suggest poor hygiene and diet deficiency, respectively. As regards the high frequency of bronchopneumonia (84%), the Balkan continental climate which dominates the weather in Albania (hot summer and very cold winter in the inland territories) may have a role in respiratory trait pathologies in debilitated children under poor housing conditions.

Sensitivity of bone marrow microscopy for *Leishmania* diagnosis (77.5%) was considerably lower than figures reported in published cases series of Mediterranean VL, both in children (97.6%) and adults (97.0%) [15], [16]. This may reflect a low degree of accuracy in performing the parasitological investigation. Starting from 1999 we could rely on commercial serological assays widely used in European hospital laboratories. Through serology, we could confirm the disease in 195/203 (96%) bone-marrow negative patients with strong clinical evidence of VL and in whom other systemic pathologies had been excluded. Only 6.4% of our patients received presumptive treatment due to the lack of specific VL diagnosis; all of them were clinically cured following pentavalent antimony therapy. As regards the possibility that *Leishmania* asymptomatic infections detected by serology were present in children exhibiting VL-mimicking conditions, it should be pointed out that differently from DAT or Western blot serological assays, or PCR methods, IFAT and ELISA commercial kits hardly detect *L.infantum* infections in children without overt clinical VL, and when they do so antibody titers are around the cut-off [17], [18]. Indeed, consultation with pediatric reference centres in developed Mediterranean countries disclosed the general consensus that presence of clinical signs and symptoms compatible with VL associated with the detection of serum *Leishmania* antibodies are established criteria for VL diagnosis alternative to *Leishmania* demonstration in bone-marrow aspirates [19].

Discovered 60 years ago, pentavalent antimonials remain the mainstay treatment of VL despite the long duration of administration and severe side effects which may be observed in some patients, especially adults with underlying conditions [20]. Meglumine antimoniate is still effective with more than 95% cure rate in those Mediterranean countries where the drug is of routine use for VL [21], whereas *Leishmania* antimony resistance is increasing in regions where massive treatments of infected dogs with antimonials, such as Italy and southern France, are common [22]. Anti-leishmanial therapy of infected pets is not a usual practice in Albania, which may explain the very high therapeutic efficacy of pentavalent antimony exhibited in our patients (99.3% cure rate). Furthermore, it appears that the drug was well tolerated in children. There were no cases of primary unresponsiveness and the relapse rate was low (0.7%). The few relapsing cases were treated successfully with a 7-day course of liposomal amphotericin B without adverse events. Treatment costs associated with this drug are not affordable by the Albanian health system, therefore we had to rely on the contribution and assistance of foreign institutions (University of Bari, Italy).

In conclusion, our study disclosed a pattern of pediatric VL which is somehow different from that of neighboring developed Mediterranean countries, despite sharing common *Leishmania* agents, reservoir and vectors: incidence of clinical disease in childhood and frequency of co-morbidities are by far more elevated, thus reinforcing the concept that poverty and leishmaniasis are strictly associated [13]. On the other hand, the study showed also important limitations, first of all a low standard laboratory diagnostic capability (as revealed by the scarce sensitivity of bone marrow microscopy and unsatisfactory border-line serological findings) associated to an over-emphasized use of presumptive treatments and *ex juvantibus* diagnosis based on clinical experience. Furthermore, the studied population may represent a biased sample of patients living in urbanized areas where the passive medical surveillance is more efficient. Therefore an improvement is warranted of a disease-specific surveillance system in Albania which includes training of laboratory staff in peripheral hospitals, active search of the infection in febrile children resistant to antibiotic therapies and the use of specific and sensitive methods for diagnosis and follow-up.
